# Graph-Based Prediction of Spatio-Temporal Vaccine Hesitancy From Insurance Claims Data

**DOI:** 10.1109/access.2025.3550775

**Published:** 2025-03-12

**Authors:** SIFAT AFROJ MOON, RITUPARNA DATTA, TANVIR FERDOUSI, HANNAH BAEK, ABHIJIN ADIGA, ACHLA MARATHE, ANIL VULLIKANTI

**Affiliations:** 1 Computational Sciences and Engineering Division, Oak Ridge National Laboratory, Oak Ridge, TN 37830, USA; 2 Biocomplexity Institute (BI), University of Virginia, Charlottesville, VA 22904, USA; 3 Department of Computer Science, University of Virginia, Charlottesville, VA 22904, USA; 4 Department of Public Health Science, University of Virginia, Charlottesville, VA 22904, USA

**Keywords:** Graph neural network, recurrent neural network, spatio-temporal problem, prediction, clustering, claim data, vaccine hesitancy, active learning, activity-based population network

## Abstract

Growing vaccine hesitancy is contributing to the decline in immunization rates for highly contagious, vaccine-preventable childhood diseases. Therefore, there has been a significant interest in understanding how hesitancy is spreading at higher spatio-temporal resolutions, enabling more targeted interventions. Motivated by this, we study the problem of prediction of vaccine hesitancy at the ZIP Code level, referred to as the VaxHesitancy problem. A significant challenge for this problem is the lack of high-resolution data that indicates hesitancy. Here, we develop a hybrid VaxHesSTL framework that combines a Graph Neural Network (GNN) and a Recurrent Neural Network (RNN) to address the VaxHesitancy problem. The GNN uses a ZIP Code-level network to capture spatial signals from neighboring areas, while the RNN models the temporal dynamics present in the data. We train and evaluate VaxHesSTL using a large dataset, namely the All-Payer Claims Databases (APCD), for Virginia, consisting of insurance claims from over five million individuals for six years. We find that an aggregated contact network or graph, developed from a detailed activity-based population network, plays an important role in the performance of VaxHesSTL, compared to graph models based solely on spatial proximity. Experiments demonstrate that VaxHesSTL outperforms a range of state-of-the-art baselines, which rely solely on historical time series data without accounting for spatial relationships. Since hesitancy data at higher spatial resolution is often unavailable or hard to get, we incorporate an active learning approach with our VaxHesSTL framework to optimize the training set without compromising the prediction performance. We find that hesitancy data for only 18% of ZIP Codes selected by active learning allows us to forecast hesitancy for all the ZIP Codes in the Virginia.

## I. INTRODUCTION

Effective vaccines exist for many highly contagious diseases, such as the MMR vaccine for measles and the IPV vaccine for polio. However, there has been a global decline in childhood immunization rates, which has only become worse after the COVID-19 pandemic [1], [2], [3]. In 2020 and 2021 alone, over 27 and 25 million children were estimated to have missed their first dose of the measles vaccine, respectively [2], [4]. As a result of reduced immunization rates, outbreaks of measles and other Vaccine Preventable Diseases (VPDs) have been occurring regularly in recent years across the world, e.g., in New York in 2019 [5]. There are several reasons behind the drop in immunization rates with *vaccine hesitancy* being the leading one [6]. Identifying regions with hesitancy and increasing immunization rates is a fundamental public health problem, since this allows public health agencies to target specific regions for intervention [3], [7].

*The focus of our paper is to develop methods to predict vaccine hesitancy at the level of the target spatial or administrative unit*, which we refer to as the VaxHesitancy problem. We model vaccine hesitancy among children aged between 0–6 years, who are expected to receive a set of mandatory vaccines, including MMR (Measles, Mumps, and Rubella), HepB (Hepatitis B), IPV (Inactivated Polio Vaccine), and DTaP (Diphtheria, Tetanus, Pertussis). One of the significant challenges in understanding the extent of hesitancy and how it is spreading is the lack of availability of high-resolution spatio-temporal data. Recently, many insights into vaccine hesitancy have been obtained from survey data, e.g., [8], [9], and [10]. However, some of these data are primarily related to the COVID vaccine [8], [9], and they are not available on an ongoing basis for mandatory childhood vaccines. Furthermore, these survey data generally have limited spatio-temporal resolution, making it difficult to determine the level of hesitancy at a fine-grained level, which is helpful for targeted public health interventions. Previous literature also uses social media data to understand vaccine hesitancy [11], [12]. However, these data contain significant biases, and limited spatial resolution, which can limit their use for the VaxHesitancy problem [13].

In this research, we offer a novel approach to solve the VaxHesitancy prediction problem for childhood vaccines using the All Payers Claims Database (APCD) for Virginia, an extensive insurance claims dataset that includes all insurance claims for over five million individuals over a six-year period (see Section IV-A for details). Each claim provides demographic information for the patient, diagnosis (specified through an ICD code [14]), provider, etc. Although vaccine hesitancy may have a broader definition and it is almost impossible to precisely quantify the proportion of the population that is vaccine-hesitant [15], we have used patient refusal in the APCD data as an indication of vaccine hesitancy. Insurance claims data has immense potential in various healthcare studies due to its digital form and anonymity preservation [16], [17]. Over the past few decades, researchers have used APCD data to address various public health questions, such as predicting future healthcare costs [18], measuring the quality of drug surveillance [19], studying specific diseases, and understanding age- and gender-related risks for diseases like diabetes [20]. Previous research confirms that APCD is a promising source of data to conduct population-based research [21].

**FIGURE 1.**
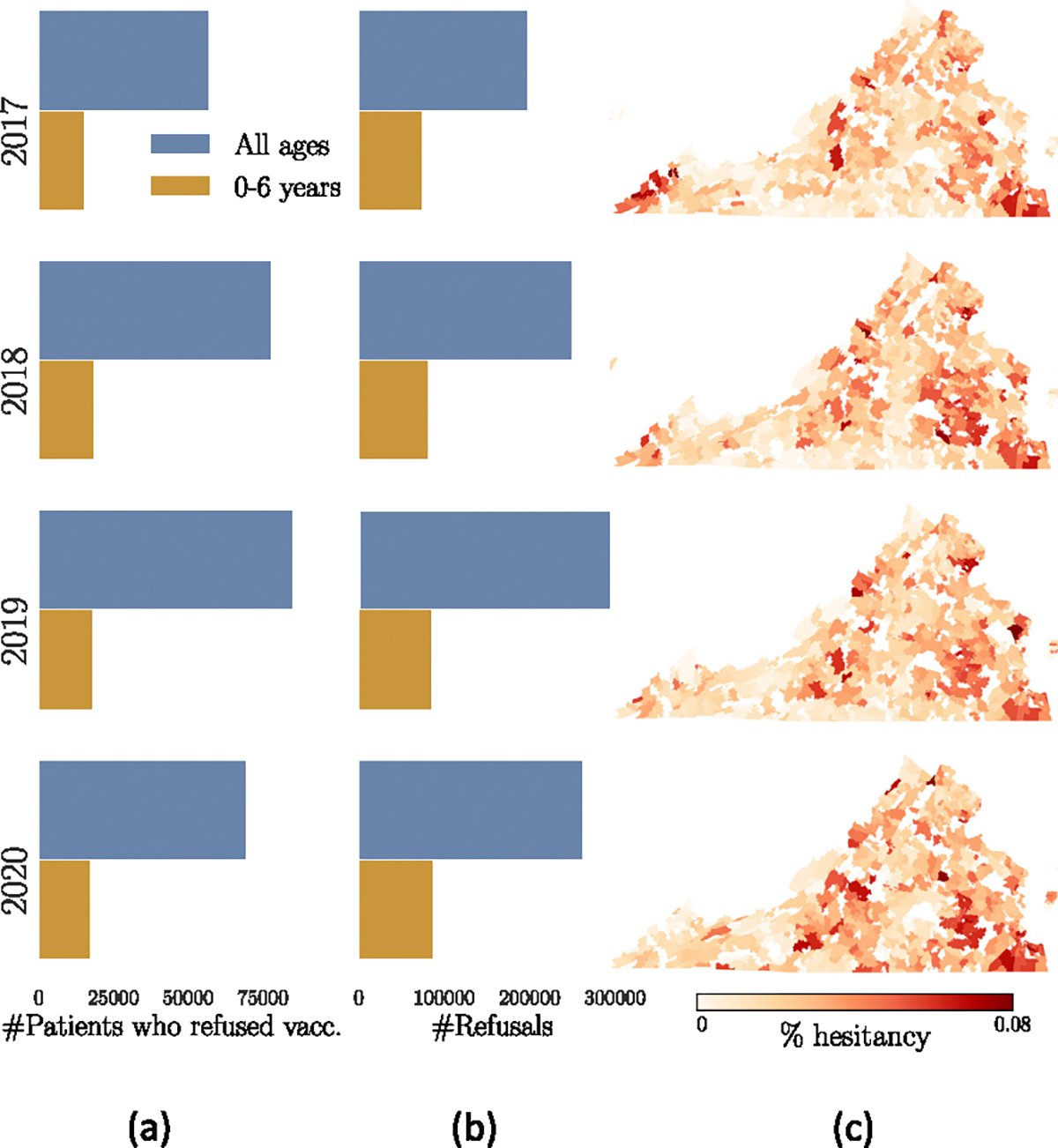
The spatio-temporal trends of vaccine hesitancy as observed in the APCD data from 2017 to 2020. Column (a) represents the number of unique patients who refused to take vaccine in a year. Column (b) presents the total number of vaccine refusal occurrence in a year. Heatmaps of yearly vaccine hesitancy at the ZIP Code level are shown in Column (c). The definition and detailed explanation of refusal and hesitancy are given in Section IV.

Analysis of APCD data reveals spatio-temporal variations in vaccine hesitancy intentions across Virginia (Figure 1). We further demonstrate spatial influence in the data by employing two statistical measures—Moran’s I and the isolation index [22]. Prior work with different datasets has also shown that vaccine refusal exhibits significant spatial correlations [23], [24], [25]. For instance, the immunization status of children in the same neighborhood, schools, or jurisdictions is often correlated. While previous modeling approaches have explored various factors influencing vaccine hesitancy, they often overlook the role of spatial relationships between communities [26], [27]. To address this gap, we focus on capturing spatial signals between ZIP Codes to improve the prediction of vaccine hesitancy. Specifically, we experiment with different connectivity schemes: aggregated activity-based contacts between populations, geographic adjacency, and distance between their centroids. By incorporating an aggregated contact network derived from a detailed, activity-based, individual-level population model [28], [29], we aim to highlight the influence of in-person interactions on vaccine hesitancy. To account for the spatial correlations, GNNs are used, a class of deep neural networks designed to handle graph data [30], [31], [32], [33]. GNNs have demonstrated good prediction capabilities for spatial data, such as house price estimation [34], understanding election results [35], and weather forecasting [36].

Research has shown that vaccine hesitancy sentiment evolves over time, influenced by various socio-political and health-related factors [6]. Capturing these temporal dynamics is crucial for accurate prediction and intervention. Prior studies have employed recurrent neural network (RNN) architectures, such as Long Short-Term Memory (LSTM) networks [37], to effectively model time-dependent behaviors in similar domains. These models are particularly adept at handling sequential data, allowing for the tracking of changes in vaccine hesitancy sentiment across different time steps.

Recent studies have utilized spatio-temporal graph learning for various forecasting tasks, such as traffic flow prediction [38] and disease prevalence estimation [39]. Prior works have employed combinations of GNNs, RNNs, and Convolutional Neural Networks (CNNs) for spatio-temporal forecasting [40], [41]. Yu et al. [40] show the potential of graph-based learning frameworks for timely and accurate traffic forecasts with comparisons between CNN and RNN. All these works illustrate the potential of integrating spatial and temporal dynamics within forecasting models, setting a foundation for similar approaches in understanding vaccine hesitancy trends across different ZIP codes. Building on these approaches, we apply a similar framework to our APCD data and achieve strong predictive performance.

Previous studies have highlighted that obtaining high-resolution data on vaccine hesitancy is often challenging or expensive [15]. Active learning has been studied to improve the efficiency of training when labeled data is limited. This research uses a prominent active learning approach to address data limitations in the VaxHesitancy study: the Active Learning for Graph Embedding (AGE) framework [42], which selects the most informative nodes for querying by leveraging graphical information such as node centrality and embedding features. AGE incorporates factors like classification uncertainty and the representativeness of node embeddings to optimize the selection process.

**FIGURE 2.**
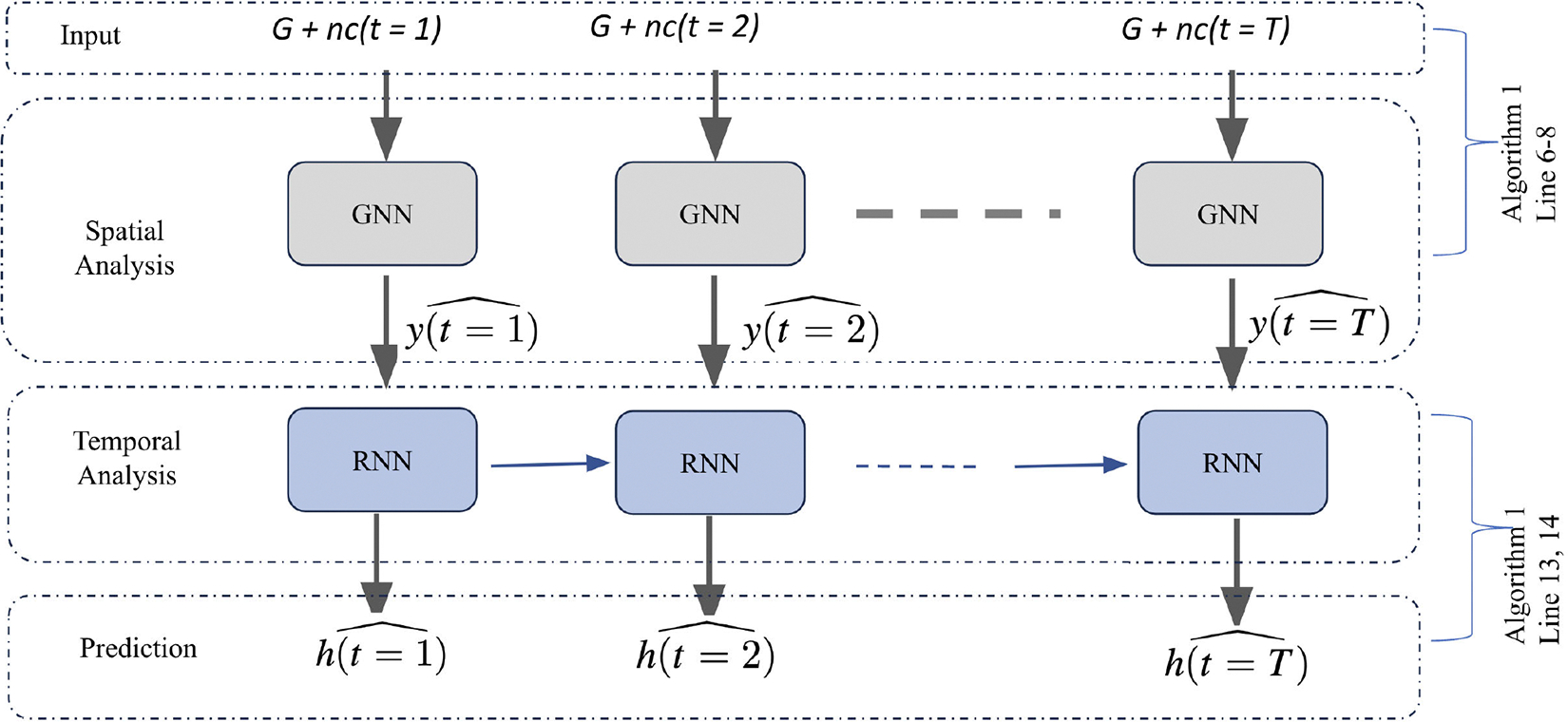
Architecture of the spatio-temporal graph-based node-level regression learning, VaxHesSTL, for the prediction of vaccine hesitancy. The input to VaxHesSTLis a graph G and a feature matrix nc(t) at time t. The output of VaxHesSTL at time t is the predicted vaccine hesitancy.Here, T represents the target time step. Algorithm 1 is presented in Section III.

Our main contributions in this research are as follows:

- We introduce a novel approach for addressing the VaxHesitancy problem: the Vaccine Hesitancy Spatio-Temporal Learning (VaxHesSTL) framework (Figure 2). It combines a Graph Neural Network (GNN) with a Recurrent Neural Network (RNN). The GNN leverages an aggregated contact network to capture the spatial aspects of vaccine hesitancy, while the RNN captures the temporal dynamics from historical hesitancy data. We train and evaluate VaxHesSTL on the APCD, and our framework outperforms several state-of-the-art (SOTA) baselines, leading to a substantial reduction in prediction errors, ranging from 6% to 64.14% compared to the SOTA baselines.
- Through an ablation study, we demonstrate the effectiveness of the combined framework VaxHesSTL in enhancing model performance, particularly emphasizing the importance of incorporating spatial information derived from detailed population-level mixing data for forecasting vaccine hesitancy.
- We identify the distinguishing population characteristics of ZIP Codes with high prediction errors. Some demographic characteristics such as the distributions of age-groups and ethnicity, and vaccine hesitancy percentage are substantially different for these exceptions compared to the rest of the regions.
- Acquiring spatio-temporal vaccine hesitancy data is often expensive or unattainable, and data gaps are prevalent in sources like insurance claims [43], [44]. We integrate *active learning* into the VaxHesSTL framework to strategically select the most informative training samples within a fixed budget. This approach leads to a performance improvement of over 15%, even when using a training set comprising less than one-fifth of all ZIP Codes. This demonstrates the significant gains from active learning in uncovering critical patterns and insights in the VaxHesitancy problem.

Our framework VaxHesSTL is flexible enough to incorporate other datasets. It can be utilized to predict vaccine hesitancy for any specific vaccine across various geographic areas, as long as spatio-temporal vaccine hesitancy data, demographic information of target population, and a connectivity graph are available. The rest of the paper is organized as follows: Section II describes the related work, Section III presents the VaxHesSTL methods and the active learning setup, Section IV describes experiments and results, and finally Section V concludes the paper.

## II. RELATED WORK

Contemporary data-driven studies explore different machine learning models to find local vaccine hesitancy hotspots or to predict individual decisions for childhood immunization [26], [27], [45]. Carrieri et al. propose a random forest machine learning (ML) algorithm to identify key area-level indicators for predicting hesitancy toward non-mandatory childhood vaccines in 6,062 Italian municipalities in 2016 [26]. This study finds that the level of waste recycling and employment rate are the most important area-level predictors of non-mandatory childhood vaccines.

While most recent studies on vaccine hesitancy focus on the COVID-19 vaccine [46], it is important to understand hesitancy toward childhood vaccines, as many of these are mandatory. Machine learning models; such as, recursive partitioning, support vector machines (SVMs), random forests, and C-forest models can identify children who are at a higher risk for defaulting on follow-up immunization visits from longitudinal immunization records [27]. The ML models can predict individual-level vaccine hesitancy effectively from individual-level electronic health record (EHR) data and other health check-up data [45]. However, these studies depend on proprietary datasets, such as siblings’ vaccination history and private medical history. Vaccine hesitancy has spatial nature that these studies do not consider.

Mollalo and Tattar [46] study the spatial distribution of vaccine rates in the US based on social vulnerability index using a multi-scale geographically weighted regression model. Their work shows that the importance of covariates varies across space. A recent study of a spatially structured mathematical model of opinion dynamics explains the occurrence of vaccine hesitancy as the result of the presence of echo chambers [47]. Ling et al. explore an optimal vaccine allocation strategy by proposing a combined framework of GNN and reinforcement learning. They also address the importance of higher-resolution mobility data in vaccine studies.

Active learning is a powerful machine learning approach, particularly useful when training data is limited. It is widely applied in fields such as natural language processing, computer vision, and bioinformatics. Wu et al. [48] enhance the AGE framework by combining node feature propagation with K-Medoids clustering, effectively identifying key instances for labeling in node classification tasks using GNNs. Additionally, graph partitioning methods [49] have been explored, focusing on selecting representative nodes from each partition to improve the efficiency of active learning in GNNs. Active learning is a useful and novel tool to address data limitations in the VaxHesitancy study.

## III. VACCINE HESITANCY PREDICTION FRAMEWORK

### A. PRELIMINARIES

We aim to learn vaccine hesitancy, measured by the percentage of 0–6-year-old patients refusing to take the vaccine within a ZIP code in APCD data. As inputs, we use historical vaccine hesitancy data and demographic features of a ZIP code (which includes age, gender, race, ethnicity, and insurance type), and spatial ZIP code level connectivity information.

Let G(V,E,W) be a graph or network, where V denotes the set of N ZIP codes or nodes, E is the set of edges, and $W\in R^{N\times N}$ is a weighted adjacency matrix. We have developed graph G from an activity-based detailed network (described below in Section III-B1). Each entry of W,w(i,j), represents the activity-based connections from ZIP code i to j. We have compared our framework with the two most common spatial connectivity in epidemic modeling as baselines: 1) geographic adjacency and 2) distance-based.

Let $nc_{i}\left(\left[t_{1},\;t_{2}\right]\right)$ and $h_{i}\left(\left[t_{1},\;t_{2}\right]\right)$ denote the time series of features and hesitancy level in ZIP code i in the time interval [$t_{1},\;t_{2}$ ], respectively; when $t_{1}=t_{2}=t$, we denote these by $nc_{i}(t)$ and $h_{i}(t)$. Let $nc\left(\left[t_{1},{\;t}_{2}\right]\right)\in R^{N\times X\times TS}$ and $h\left(\left[t_{1},\;t_{2}\right]\right)\in R^{N\times TS}$ denote the corresponding N nodes, here, X is the number of features, and TS is the number of time steps from $t_{1}$ to $t_{2}$. We use ${nc}_{K}\left(\left[t_{1},{\;t}_{2}\right]\right)$ and $h_{K}\left(\left[t_{1},\;t_{2}\right]\right)$ to represent features and hesitancy levels for a subset of nodes, where K⊂V.

Let $F\left(j,t,G,{nc}_{k}\left(\left[t_{1},t_{2}\right]\right),h_{k}\left(\left[t_{1},t_{2}\right]\right)\right)$ be a function that predicts $h_{j}(t)$, using $G,\;{nc}_{k}\left(\left[t_{1},t_{2}\right]\right)$ and $h_{k}\left(\left[t_{1},t_{2}\right]\right)$ as inputs. This paper focuses on addressing the following prediction challenges:

1. **The spatio-temporal vaccine hesitancy prediction problem**, VaxHesitancy (K): For a subset K⊆V, find a function $F\left(j,T+1,G,{nc}_{K}([1,T]),h_{K}([1,T])\right)$ for all j∈V.
2. **Active learning for vaccine hesitancy prediction**, VaxHesitancyAL (B): Choose a subset $K^{\prime }$ with a fixed budget B, such that $F\left(j,T+1,G,{nc}_{K^{\prime }}([1,T])\right.,\;\left.h_{K^{\prime }}([1,T])\right)$ for $\left.j\in V-K^{\prime }\right)$, maximizes accuracy.

The notations used throughout the paper are summarized in the Table 1.

### B. *VAXHESSTL* FRAMEWORK

Analysis of the APCD dataset reveals spatio-temporal heterogeneities and correlations in vaccine hesitancy levels (Figure 1), which motivates our GNN-RNN approach VaxHesSTL. Our framework comprises (i) a spatial module and (ii) a temporal module (Figure 2).

#### 1) SPATIAL MODULE *M*_*S*_

##### a: GRAPH ARCHITECTURE

**FIGURE 3.**
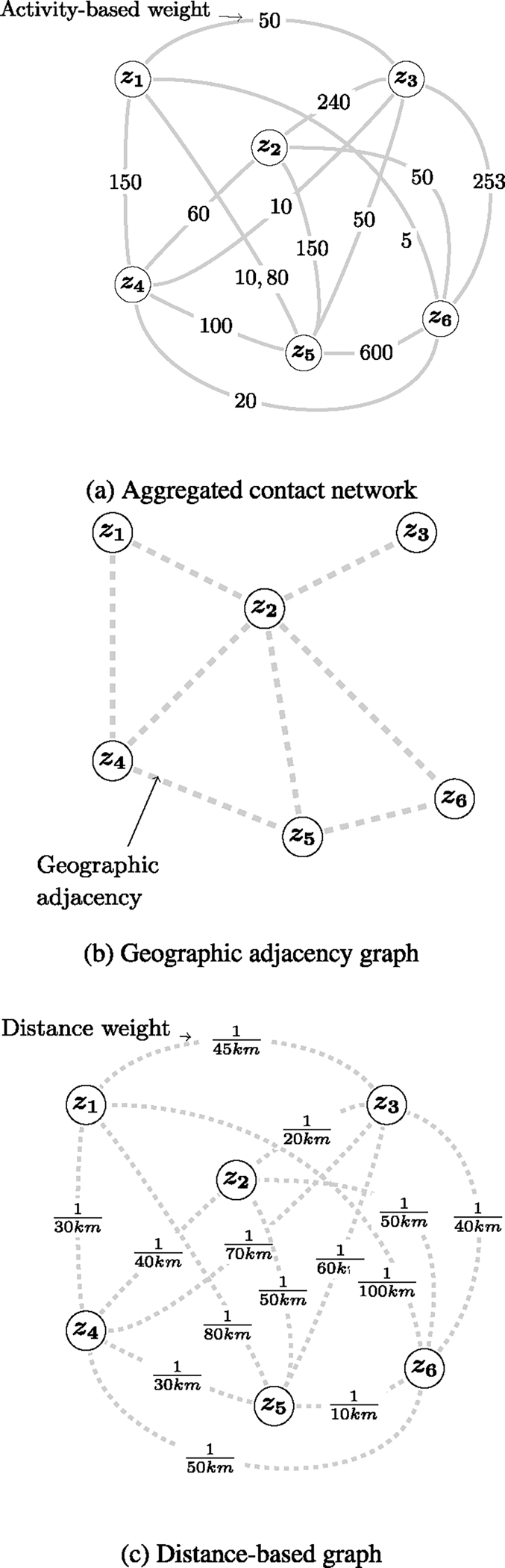
Variants of Graph G. (a) Aggregated contact graph $G_{a}$, (b) geographic adjacency graph $G_{b1}$, and (c) distance-based graph $G_{b2}$.

The graph G is a static graph without self-loops. We propose three intuitive node connectivity mechanisms to form three variants of G (Figure 3).

- Aggregated contact network, $G_{a}$: This graph represents human activity contacts between two ZIP codes. If m physical contacts occur between the populations of ZIP codes i and j, and each contact has an activity weight a, then the graph $G_{a}$ will have an edge between ZIP codes i and j with weight $\sum_{i=1}^{m}\;a_{i}$. Details of construction are present in Section IV-A.
- Geographic adjacency graph, $G_{b1}$: If node i and node j share a geographic boundary, they will form an edge in the network $G_{b1}$. The weight on each edge is 1.
- Distance-based graph, $G_{b2}$: This is a fully connected graph where the weight on edge (i,j) has a weight equal to the inverse of the distance between the centroids of the two endpoints.

All edge weights are normalized using min-max normalization. In Figure 3, we show three types of graphs where the edges are weighted according to the categories mentioned above.

##### b: GRAPH-BASED SPATIAL DEPENDENCY LEARNING

Exploring the spatial distribution of vaccine hesitancy with demographic characteristics is a major task. We leverage graph neural network (GNN) [50] to learn spatial dependency for each time step through message passing. In this work, node features change with time, but the graph connectivity is static. For each time step, we have one GNN module, which consists of stacking multiple GraphConv GNN layers and a linear layer to perform node-level vaccine hesitancy prediction. Morris et al. [32] develop GraphConv, which proposes a generalization of GNNs, k-GNNs, based on the k-dimensional Weisfeiler-Leman algorithm (k-WL). We use the GNN module for a node-level regression task. In all of our experiments, we have used k=1. The output of the graph embedding module is an intermediate solution vector $\hat{y(t)}$ for a time step t.

##### c: LOSS FUNCTION FOR SPATIAL MODULE, LOSS_1_

We optimize this module using an Adam optimizer and the Mean Absolute Percentage Error (MAPE) loss function to minimize the error in graph learning between the predicted value, $\hat{y(t)}$, and the target vaccine hesitancy, y(t). We calculate the loss for a time step t=0 for the training set node K⊆V, as

(1) $$loss_{1}=\mathrm{MAPE}(y_{K}(t)-\hat{y_{K}(t)})+\lambda L_{r}$$

Here, λ is a hyper-parameter, and $L_{r}$ is the L2 regularization term to prevent over-fitting. We consider $y_{K}(t)=h_{K}(t)$ to optimize spatial module.

#### 2) TEMPORAL MODULE $M_{T}$

To learn the temporal aspect of the vaccine hesitancy for a node i, we use a temporal module. This module can be built using any model that can learn sequences; popular choices are moving average, Autoregressive Integrated Moving Average (ARIMA), and Recurrent Neural Networks (RNN). RNN is a well-known AI technique for predicting sequence data. In this work, we use the Long Short-Term Memory (LSTM) variant of RNN [37]. LSTM employs gated mechanisms to retain extensive long-term information and it performs better in the VaxHesSTL framework for the VaxHesitancy problem than other RNNs.

##### a: LOSS FUNCTION FOR TEMPORAL MODULE, LOSS_2_

We optimize this module using another Adam optimizer and the same error metric, MAPE, to minimize the error in time sequence between the predicted value, $h\hat{\left([1,t]\right)}$ and the target vaccine hesitancy, h([1,t]).

(2) $$loss_{2}=\mathrm{MAPE}(h_{K}([1,t])-\hat{h_{K}([1,t}]))+\lambda L_{r}$$

Here, again λ is a hyper-parameter, and $L_{r}$ is the $L_{2}$ regularization term. We optimize two modules separately as we do not want to influence one module’s parameters due to the other module’s performance. Optimization of two modules at the same time with one loss function does not perform well for our objective and dataset.

**Algorithm 1:** VaxHesSTL for Spatio-Temporal Vaccine Hesitancy Learning and Evaluation: $F\left(j,T+1,G,{nc}_{K}([1,T])\right.$ for All j∈V

Input: Feature matrix nc, graph G, Spatial module $M_{s}$, temporal module $M_{t},\;\lambda$, number of training steps ${train}_{s}$, hyper-parameters	
**Output:** Predicted hesitancy at time T+1 for ZIP code V-K set, $\hat{h_{V-K}K(T+1)}$	
1:	*Training Set* $\leftarrow nc_{K}[1,T]$, $h_{K}[1,T]$, K⊆V
2:	Initialize model $M_{s}$ and $M_{t}$ with hyper-parameters
3:	Set optimizer with hyper-parameters
4:	Set $M_{s}$ and $M_{t}$ in the training mode
5:	**for** number of training steps ${train}_{s}$ **do**
6:	**for** each time step t **do**
7:	**if** t<T+1 **then**
8:	$\hat{y_{K}(t)}\leftarrow M_{s}\left(G,nc_{K}[t]\right)$ {Train $M_{S}$ with *Training Set*}
9:	Compute ${loss}_{1}=MAPE(y_{K}(t)-\hat{y_{K}(t)})+\lambda L_{r}$
10:	Update $M_{s}$ using *Adam* optimizer
11:	**end if**
12:	**end for**
13:	$\left.\hat{h_{K}(\left[1,T\right]}\right)\leftarrow M_{t}(\hat{y_{K}(\left[1,T\right]}))$
14:	Compute ${loss}_{2}=MAPE(h_{K}([1,T])-(\hat{h_{K}(\left[1,T\right]})+\lambda L_{r}$
15:	Update $M_{t}$ using *Adam* optimizer
16:	**end for**
17:	Set $M_{s}$ and $M_{t}$ in the evaluation mode
18:	**while** t<T+1 do
19:	$\hat{y_{V-K}(t)}\leftarrow M_{s}\left(G,nc_{V-K}[t]\right)$
20:	**end while**
21:	Form $Y_{V-K}$ from all $\hat{y_{V-K}\left(\left[1,T\right]\right)}$
22:	$\hat{h_{V-K}\left(T+1\right)}\leftarrow M_{t}(\hat{y_{V-K}\left[1,T\right]})$
23:	**return** $\hat{h_{V-K}\left(T+1\right)}$

### C. ACTIVE LEARNING

Data gaps exist that impede predicting of vaccine hesitancy at a higher spatio-temporal resolution [43], [44]. Motivated by this issue, we consider an active learning scenario where relatively few ZIP codes are observed (K≪|V|) and the goal is to identify additional B ZIP codes to query for vaccine hesitancy data so that the prediction error of the learning algorithm is minimized. Here, B is the budget that is determined by resource constraints to collect this additional information.

Our framework is designed to forecast vaccine hesitancy, even when we only have access to a subset of ZIP code data, as outlined in challenge 1. However, we’ve developed a solution that’s both cost-effective and efficient for cases where our budget restricts us to gathering data for only a handful of ZIP codes. This is particularly important because acquiring claims data and vaccine hesitancy information for all ZIP codes can be challenging and resource-intensive.

We begin by selecting a small subset K of ZIP codes uniformly at random. Subsequently, we iteratively choose the remaining ZIP codes for training using a query policy that ensures diversity in the output space while staying within the budget constraint B. This approach is similar to Wu et al.’s [51] Active Learning for Regression (ALR) algorithm, where they select the most beneficial samples to label instead of random selection. The details are in Algorithm 2.

**Algorithm 2:** Active Learning for Training Set Generation for VaxHesSTL

**Input:** Feature matrix nc, Graph G, VaxHesSTL module, and Budget B	
**Output:** Training Set, $K^{\prime }$	
1:	Randomly chosen ZIP codes set, K⊂V
2:	$K^{\prime }=K$
3:	**for** $|K{|}^{\prime }\leq |K|+B$ **do**
4:	${\mathrm{VaxHesSTL}}_{K^{\prime }}\leftarrow \mathrm{VaxHesSTL}\left(G,nc_{K^{\prime }}([1,T])\right)$
	{Training VaxHesSTL with $K^{\prime }$ ZIP codes}
5:	**for** each node $x\in V-K^{\prime }$ **do**
6:	Evaluate the absolute distance, $D\left(x,k^{\prime }\right)=\|{\mathrm{VaxHesSTL}}_{K^{\prime }}\left(G,nc_{x}([1,T])\right)-h_{K^{\prime }}(T+1)||$
7:	**end for**
8:	Identify the node with the highest absolute distance, $x^{\prime }={\mathrm{argmax}}_{x}\left(D_{xk^{\prime }}\right)$
9:	Add $x^{\prime }$ to the training set $K^{\prime }$
10:	**end for**
11:	**return** $K^{\prime }$

In addition, we explore two alternative strategies for selecting the initial training ZIP codes K: scattered and closer.

- In the scattered approach, nodes (in our case, ZIP codes) are selected in such a way that they are distributed across the graph $G_{a}$, to achieve wide spatial coverage. Rather than focusing on nodes in close proximity, the scattered approach intentionally chooses nodes from different parts of the graph, ensuring that they are spread out to cover distinct areas. This selection is typically based on weighted connections in the graph.
- In the closer approach, ZIP codes are selected based on their proximity with edge weights within the graph $G_{a}$. This method focuses on choosing nodes that are closely connected, prioritizing those connected by higher-weighted edges. After selecting an initial node, subsequent nodes are chosen sequentially from among those that are directly linked to the previously selected nodes, ensuring that the selections remain within a localized area of the graph.

However, for our specific dataset, employing a *random selection strategy* prior to active learning consistently enhances accuracy. The other approaches and corresponding results are explained in the Appendix C–B.

## IV. EXPERIMENTS AND RESULTS

Our empirical analysis is motivated by the following questions.

1. How well can our model forecast vaccine hesitancy (Section IV-D)?
2. How can we achieve a reliable prediction when we have limited information available (Section IV-E)?

### A. DATA

We use an activity-based data-driven detailed network [28], [52] and an insurance claim database, All-Payer Claims Database (APCD), to study the VaxHesitancy problem.

#### 1) ACTIVITY-BASED DIGITAL TWIN INDIVIDUAL-LEVEL NETWORK MODEL *H* FOR THE VIRGINIA POPULATION

We form the aggregated contact graph $G_{a}$ from an activity-based detailed population-level social contact network $H=\left(V_{H},E_{H}\right)$ derived from a realistic representation of the US population [29], [53]. Nodes $v\in V_{H}$ in this network represent individuals in a population. Each edge $(u,v)\in E_{H}$ represents a co-location, and is associated with a weight that is equal to the duration of the contact between two individual. This network model combines various datasets from commercial and public sources, including LandScan [54], OpenStreetMap [55], the American Time Use Survey [56], the Multinational Time Use Study [57], the National Household Travel Survey [58], and US Census data, to develop a unified framework for generating a digital twin of the Virginia population. To construct H, daily activities are initially assigned to individuals within a household based on activity and time-use surveys. The locations of these activities are estimated using methods from transportation science and detailed land use data. The resulting activity-based population network model H is statistically equivalent to the census [28], [29], [53], [59].

We aggregate the population network H to get the ZIP code level aggregated contact network or graph $G_{a}$. We use ZIP code as our spatial resolution because it is the highest spatial resolution available from the APCD data. In our work, we utilize ZIP codes derived from ZCTAs. A ZCTA corresponds to a geographical representation of a service area for a United States Postal Service (USPS) ZIP Code [60]. In our framework, we only consider ZIP codes with a larger population size of more than 1000 people, which covers approximately 52% ZIP codes and 97% population of Virginia.

#### 2) ALL-PAYER CLAIMS DATABASE (APCD) OF VIRGINIA HEALTH INFORMATION (VHI)

APCD data contains paid medical and pharmacy claims for approximately 5 million Virginia residents with commercial, Medicaid, and Medicare coverage. The APCD covers almost 65% of the Virginia population over a six year period from January 1, 2016, to December 31, 2021. Each claim in the APCD has 113 fields, including a unique ID, and is associated with a service date or incurred date, detailed demographic information about the patients, patient ZIP code, service provider’s ZIP code, and service description. Table 2 lists some of the key features we use in our study.

We filter all patient claims of children aged six or below; we assume that patient refusal at this age represents parental vaccine hesitancy. We classify the “Patient refusal” entries using the International Classification of Disease ICD-10-CM code Z28, which corresponds to “Immunization not carried out and underimmunization status” [14]. After extracting the features as mentioned in Table 2, we apply a one-hot encoder to transform categorical features into columns. To prepare the dataset for a given time step t in ZIP code i, we record the number of unique children, the number of unique children by gender, race, and medical insurance type, as well as the percentage of unique children who have refused at least one vaccine. The percentage of unique children who refuse to take any vaccine at least once is the target feature h(t). We use “medical insurance type” as a proxy for the income level. In this research, we use three months as the time unit. We find that vaccine hesitancy data at a higher time resolution, such as monthly, is very sparse at the ZIP code level.

Finally, we use min-max normalization to normalize all columns for each time step t. In addition, we do Principal Component Analysis (PCA) to find the principal components of the features.

### B. MOTIVATION OF *VAXHESSTL* FROM DATA

Here, we provide a preliminary analysis of the APCD data that motivates the need for vaccine hesitancy prediction as well as our approach to the VaxHesitancy problem.

Firstly, we notice that vaccine hesitancy is increasing in Virginia in the APCD data, and this trend is confirmed by the publicly available SchoolVaxView, a CDC platform that provides vaccination coverage data for school-aged children [61]. SchoolVaxView reports that non-medical exemptions among kindergartners are increasing in Virginia. From the 2015–2016 to the 2023–2024 school year, the percentage of non-medical exemptions among kindergartners in Virginia has increased by more than 130%. Each school year has seen an increase or remained equal compared to the previous school year, except for the 2020–2021 school year. Similar to Virginia, non-medical exemptions among kindergartners are increasing in many other states in the USA, such as Alabama, Arizona, and Arkansas.

From an exploratory analysis of the APCD data, we observe that patient refusal and vaccine hesitancy exhibit both spatial and temporal variations. From the spatial analysis, we observe that vaccine hesitancy of a location has correlation with its neighboring areas (Figure 1). We use Moran’s-I and isolation index to confirm the spatial influence in the data [22].

**Moran’s-I** is a measure of global spatial autocorrelation. This value ranges from −1 to 1, with 0 indicating no autocorrelation; −1 indicating perfect clustering with dissimilar values, such as clustering of high vaccine hesitancy location with the low vaccine hesitancy location; and 1 indicating perfect clustering with similar values, such as clustering of high vaccine hesitancy locations with high vaccine hesitancy locations. The equation for the Moran’s-I for a time t is

(3) $$\begin{array}{ll} \frac{N}{\sum_{i}\;\sum_{j}\;w(i,j)}\\ & \frac{\sum_{i}\;\sum_{j}\;w(i,j)\left(h_{i}(t)-\frac{1}{N}\sum h(t)\right)\left(h_{j}(t)-\frac{1}{N}\sum h(t)\right)}{\sum_{i}\;{\left(h_{i}(t)-\frac{1}{N}\sum h(t)\right)}^{2}} \end{array}$$

The calculated Moran’s I from the $G_{a}$ network is 0.4580, which represents individuals exhibiting higher levels of vaccine hesitancy who are more likely to be situated closely in $G_{a}$.

**Isolation Index** indicates the level of segregation within a specific group or cluster compared to the larger population, with values ranging from 0 (no segregation) to 1 (full segregation). The equation for the isolation index for a time t is

(4) $$\sum_{i=1}^{N}\left[\left(\frac{h_{i}(t)p_{i}(t)}{\sum_{i=1}^{N}\;h_{i}(t)p_{i}(t)}\right)h_{i}(t)\right]$$

The calculated isolation index is 0.0480, which indicates almost no segregation.

**FIGURE 4.**
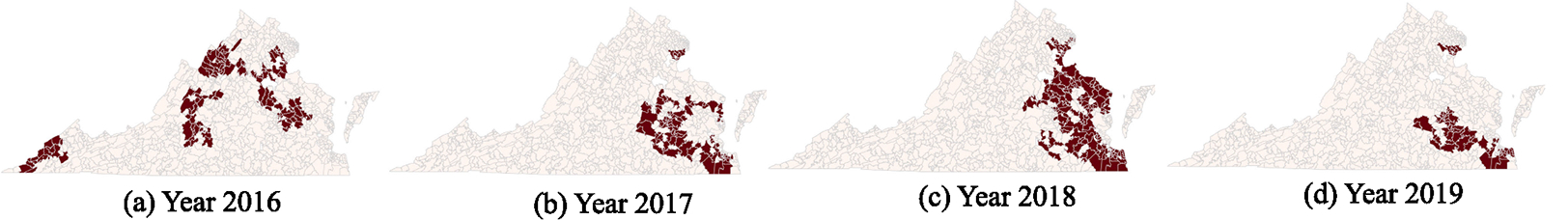
Clusters of higher vaccine hesitancy regions in Virginia in the APCD data.

Finally, we use a **network scan statistics** method to identify statistically significant geographical clusters of higher vaccine hesitancy in the data [53], [62]. The scan statistics method is a hypothesis-testing based approach for anomaly detection, used in previous studies to detect hotspots and anomalies in spatial distributions [63]. We use a Poisson version of the Kulldorff scan statistic to find statistically significant clusters of ZIP codes with higher vaccine hesitancy in the adjacent graph $G_{b1}$, using the methods from [53]; this assumes that the observations are generated from Poisson distribution. The statistically significant clusters of higher vaccine hesitant ZIP codes are presented in a dark color in Figure 4. We find that the cluster shapes and sizes change over time.

### C. SETUP AND EVALUATION

In the experiment, we use PyTorch Geometric GraphConv as GNN layer. For module $M_{g}$, we experiment on 1, 2, 3, and 4 GNN layers with the hidden units 64, 128, 256, and 512. For module $M_{t}$, we experiment on hidden units 8, 16, 32, and 64. For all GNN layers and LSTM layers, we implement 50% dropout to avoid over-fitting. We use a learning rate of 5*e* – 4, a training epoch of 15,000, and an Adam optimizer with a weight decay of 5*e* – 4 for both $M_{g}$ and $M_{t}$ modules. We use Python 3.8 to implement the framework. We utilize the open-source deep-learning framework PyTorch version 2.0.0 and NVIDIA CUDA 11.4.2 in a Simple Linux Utility for Resource Management (SLURM) system.

#### 1) EVALUATION METRICS

The focus of the VaxHesitancy study is a **node-level regression** task. We use mean absolute percentage error (MAPE), Mean Squared Error (MSE), Root Mean Squared Error (RMSE), Mean Absolute Error (MAE), and R-squared (R^2^) metrics to evaluate the performance of the VaxHesSTL framework in predicting vaccine hesitancy at the node level. We use *R*^2^ to show how well the data fit our regression model by measuring the proportion of total variance explained by the model.

- Mean Absolute Percentage Error (MAPE) measures the average error magnitude in model predictions, especially valuable in forecasting as it tells how much the predictions deviate from the actual values on average, in percentage terms.
(5) $$\text{MAPE}=\frac{1}{|\text{test}|}\sum \left|\frac{h_{\text{test}}(t)-\hat{h_{\text{test}}(t)}}{h_{\text{test}}(t)}\right|$$
- Mean Squared Error (MSE) assesses regression models by calculating the average of the squared discrepancies between observed and predicted values and offers insights into outliers and imbalances present in the dataset.
(6) $$\mathrm{MSE}=\frac{1}{|\text{test}|}\sum {\left(h_{\text{test}}(t)-\hat{h_{\text{test}}(t)}\right)}^{2}$$
- Root Mean Squared Error (RMSE), like MSE, is applied in regression task, with reduced outlier influence, yet maintaining its vital role in outlier detection within models.
(7) $$RMSE=\sqrt{\frac{1}{\left|test\right|}\sum {\left(h_{\mathrm{test}}(t)-\hat{h_{\mathrm{test}}(t)}\right)}^{2}}$$
- Mean Absolute Error (MAE) calculates average absolute distances, exhibiting robustness towards outliers than MSE and RMSE.
(8) $$MAE=\frac{1}{|\mathrm{test}|}\sum \left|h_{\mathrm{test}}(t)-\hat{h_{\mathrm{test}}(t)}\right|$$

Only MAPE is used during model training. For the test data set, MSE,RMSE and MAE metrics are also utilized to gauge the model’s predictive performance. Smaller values of the above mentioned metrics indicate better prediction accuracy.

#### 2) BASELINE METHODS

The performance of our combined graph framework VaxHesSTL is compared with the following baseline methods,

- **Linear Regression with Neighbors (LRN):** We evaluate our model’s performance against the linear regression approach. Since this method does not account for spatial correlation, to make a fair comparison, we provide an extra feature, neighbor’s information. For each node i, we calculate $\sum_{j\in 𝒩(i)}h_{j}(t)a(i,j)$.
- **Multi-layer Perceptron (MLP):** This feedforward neural network architecture is capable of capturing complex non-linear relationships within data.
- **Graph Neural Network & Gated Recurrent Units (GNN-GRU):** GNN-GRU is our third benchmark method. We replace LSTM with GRU [64] which has a simpler architecture with fewer parameters and is designed to capture long-range dependencies in sequential data.
- **Graph Convolutional Network & Long Short-Term Memory (GCN-LSTM):** In this fourth benchmark, we replace $M_{t}$ module with the GCN [30] in the VaxHesSTL [40].

### D. PERFORMANCE OF *VAXHESSTL*

The setup for $M_{g}$ with 246 hidden units and $M_{t}$ with 32 hidden units performs better for the VaxHesSTL. We find that using more than two GNN layers overfits the training data while using less than two introduces bias in the system. We also observe that separate optimizers for $M_{g}$ and $M_{t}$ give us better performance as our data size is smaller than other familiar applications, such as traffic forecasting [40]. However, having a smaller dataset is typical in the public health domain.

#### 1) IMPORTANCE OF NETWORK STRUCTURE

**FIGURE 5.**
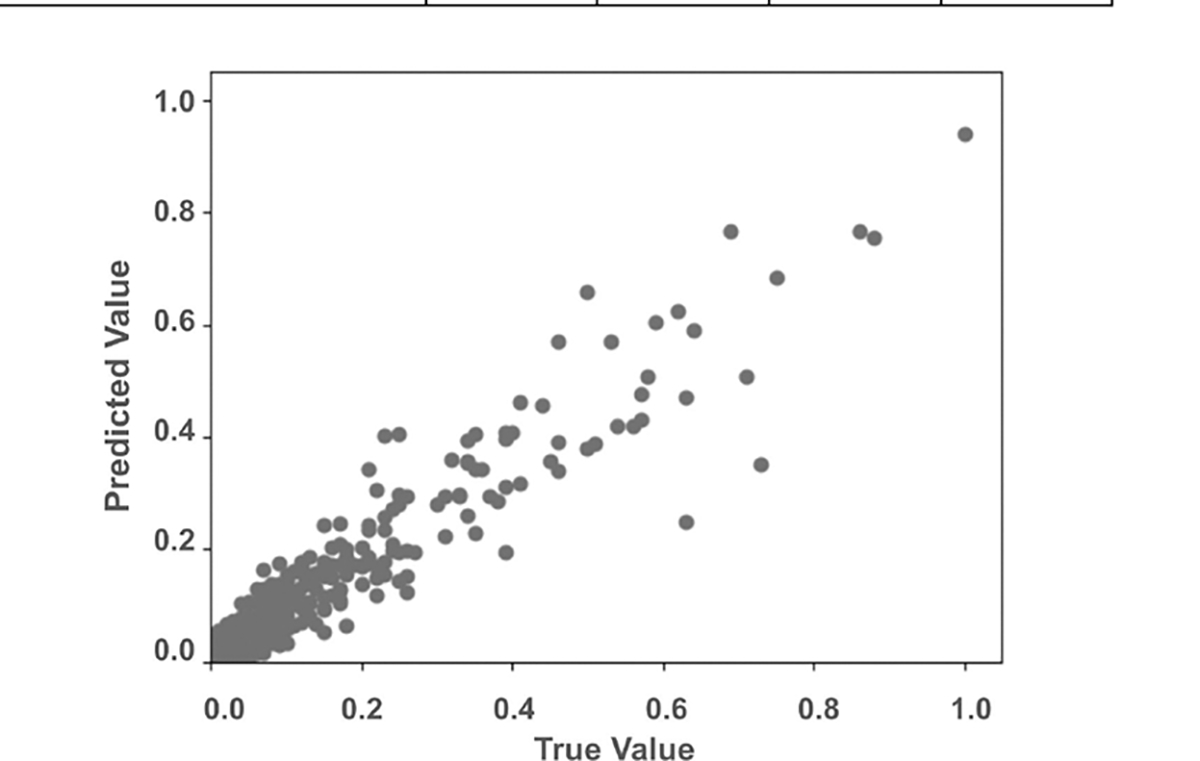
Expected predicted vaccine hesitancy and the true vaccine hesitancy in the *test* set for the final quarter of the year 2021.

The performance of VaxHesSTL depends on the right choice of node connectivity mechanisms. Table 3 shows the performance of VaxHesSTL using three G variants for 2021. The VaxHesSTL performs the best when aggregated contact graph $G_{a}$ is used. On the other hand, the geographic adjacency graph $G_{b1}$ performs the worst, suggesting that neighbors with higher activity-based connections provide a stronger spatial signal for predicting vaccine hesitancy than simple geographically adjacent neighbors. Hence, the remaining set of results in this paper for VaxHesSTL is produced using $G_{a}$. In Figure 5, the predicted values for T+1 timestep are plotted against the true values for the normalized quarterly vaccine hesitancy data of 2021, resulting in an expected *R*^2^ value of 0.87.

#### 2) PERFORMANCE COMPARED TO BASELINE METHODS

Table 4 shows the mean performances of all the baselines compared to the VaxHesSTL framework for the target time step for 2020 and 2021. In this analysis, we made predictions for the T+1 and *T* +4 time steps while training happens until T. The same *train* and *test* data sets were used for all models. We always use a batch gradient process to update the model parameters. We employ model-specific hyperparameters to unlock their full potential. Results indicate that VaxHesSTL outperforms all baselines in all evaluation metrics.

In the VaxHesSTL framework, we use GraphConv, while for GCN-LSTM, we use PyTorch Geometric’s GCNConv. The performance of GCNConv is worse compared to GraphConv in predicting hesitancy due to its design and neighbor aggregation approach. Specifically, GCNConv treats self-connections and edges to neighboring nodes with equal importance [30], which can lead to less effective feature aggregation. In contrast, GraphConv uses a K-hop neighborhood-based approach, which allows for a more nuanced and localized aggregation of node features [32]. This difference in aggregation strategy is why the GNN model GraphConv performs better in our case.

#### 3) ABLATION STUDY

We conduct an ablation study to understand the impact of two modules of the VaxHesSTL framework, $M_{s}$ and $M_{t}$.

- **VaxHesSTL w/o** $M_{t}$ **module:** In this setup, we only train the spatial learning module $M_{s}$ with the ${loss}_{1}$ function and evaluate the prediction performance only using trained $M_{s}$.
- **VaxHesSTL w/o** $M_{s}$ **module:** In this setup, we only keep the temporal learning module, which is the LSTM model. Here, we train $M_{t}$ for t=1 to t=T and test for t=T+1 time step. It does not consider any graph structure.

The last two rows of Table 4 show ablation study results. Ablation study shows the importance of spatial learning for node-level vaccine hesitancy forecasting. Although our combined framework performs better than either of these configurations, the VaxHesSTL w/o $M_{t}$ outperforms VaxHesSTL w/o $M_{s}$, indicating the importance of the spatial signal in explaining vaccine hesitancy.

#### 4) PERFORMANCE CONSISTENCY OF VAXHESSTL

We verify the VaxHesSTL’s consistency by training it on a fixed time window and making predictions at various future time steps to assess stability across different time points. We fix a training window $t^{\prime }$ and train VaxHesSTL with t=0 to $t=t^{\prime }$ and predict one step ahead of vaccine hesitancy. We then compare this with another model trained from t=1 to $t=1+t^{\prime }$, predicting its future hesitancy. Table 5 shows that, for a fixed window $t^{\prime }$, the forecasting performance across all models remains comparable.

#### 5) PERFORMANCE ANALYSIS AT THE NODE LEVEL

We investigate the properties of nodes where VaxHesSTL exhibits poor performance. For a comparative analysis, we divide the nodes into two sets: one where VaxHesSTL performs poorly and the other where it performs well. To achieve this, we first sort the nodes based on the absolute error between predicted and true vaccine hesitancy values, then divide the nodes as follows:

- **Nodes with Large Error,** $V_{L}$: Top 25% nodes from the sorted list, nodes with large error, where VaxHesSTL did not perform well.
- **Nodes with Small Error,** $V_{S}$: Rest of the test nodes, where VaxHesSTL performs well comparatively.

We investigate features of two sets: $V_{L}$ and $V_{S}$, to understand why the VaxHesSTL framework does not predict well in $V_{L}$ set. We find that population sizes, vaccine hesitancy percentages, population percentages with Medicaid insurance, and Hispanic populations differ between these two sets. Table 6 reports the average of these features for $V_{L}$ and $V_{S}$ sets. Table 6 shows that the children’s population sizes differ significantly between $V_{L}$ and $V_{S}$, indicating that VaxHesSTL tends to have larger predictive errors for nodes with smaller population sizes.

#### 6) PERFORMANCE ANALYSIS WITH STGCN

We extend our experiment by exploring additional frameworks commonly used for spatio-temporal data and prediction tasks. Among these, the most relevant is STGCN, which integrates graph structures with node attributes and applies a CNNs (Convolutional Neural Networks) model for forecasting [65]. In the Appendix, we outline how we processed our APCD data and divided it into multiple observations for training purposes 3. We also illustrate its framework in the Appendix, Figure 7. This approach enables accurate forecasting for future timesteps. We get an *R*^2^ value of 0.892 in forecasting future timestep, when STGCN is trained with data from all ZIP codes, which indicates its efficacy in addressing Problem 1 when K=V. However, STGCN is limited in predicting outcomes for unknown ZIP codes based on information from a subset of ZIP codes K, where K⊂V. This limitation arises because its temporal component processes input first before passing it to the spatial unit. Consequently, if data from certain ZIP codes are missing, the model struggles to predict effectively.

Our proposed VaxHesSTL consistently outperforms STGCN in scenarios with incomplete data, a common challenge in public health. Its robust performance under these conditions, coupled with its flexible structure that supports active learning, highlights its suitability for real-world applications in predicting vaccine hesitancy.

### E. PERFORMANCE ANALYSIS WITH ACTIVE LEARNING

The active learning framework begins by using a limited number of labeled data points, denoted as K, to initially train the VaxHesSTL model. Afterward, within a designated label budget B, ZIP codes are selected iteratively to enhance the diversity of the model. This results in a final training set size of $|K^{\prime }|\;=\;|K|\;+\;B$ for the VaxHesSTL.

**FIGURE 6.**
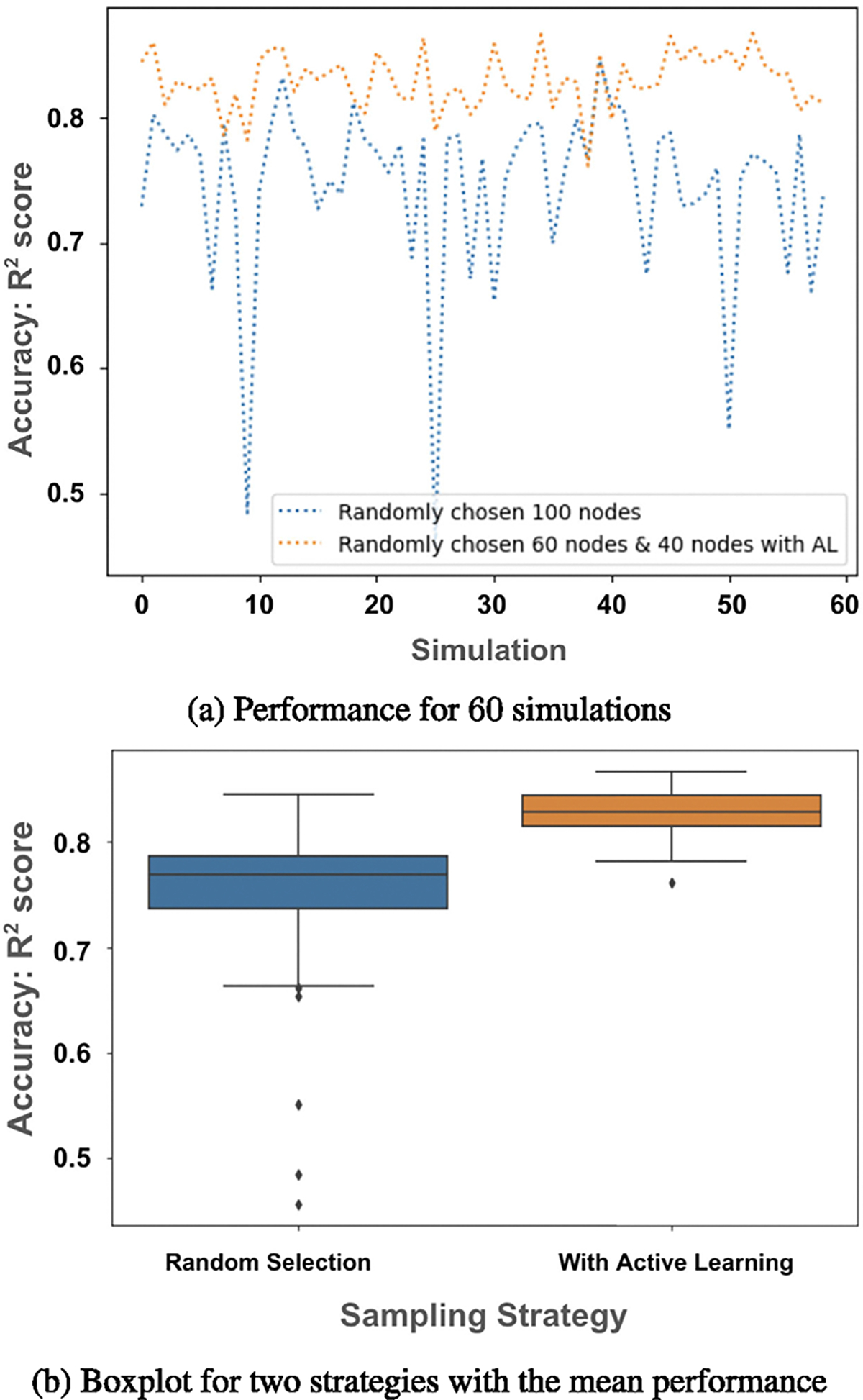
The performance of VaxHesSTL, with and without active learning, was evaluated across 60 simulations with random seeds. Subplot (a) demonstrates that VaxHesSTL achieves higher *R*^2^ values when active learning is employed. Subplot (b) shows the distribution of *R*^2^ values in the performance of VaxHesSTL in the cases with and without active learning.

**FIGURE 7.**
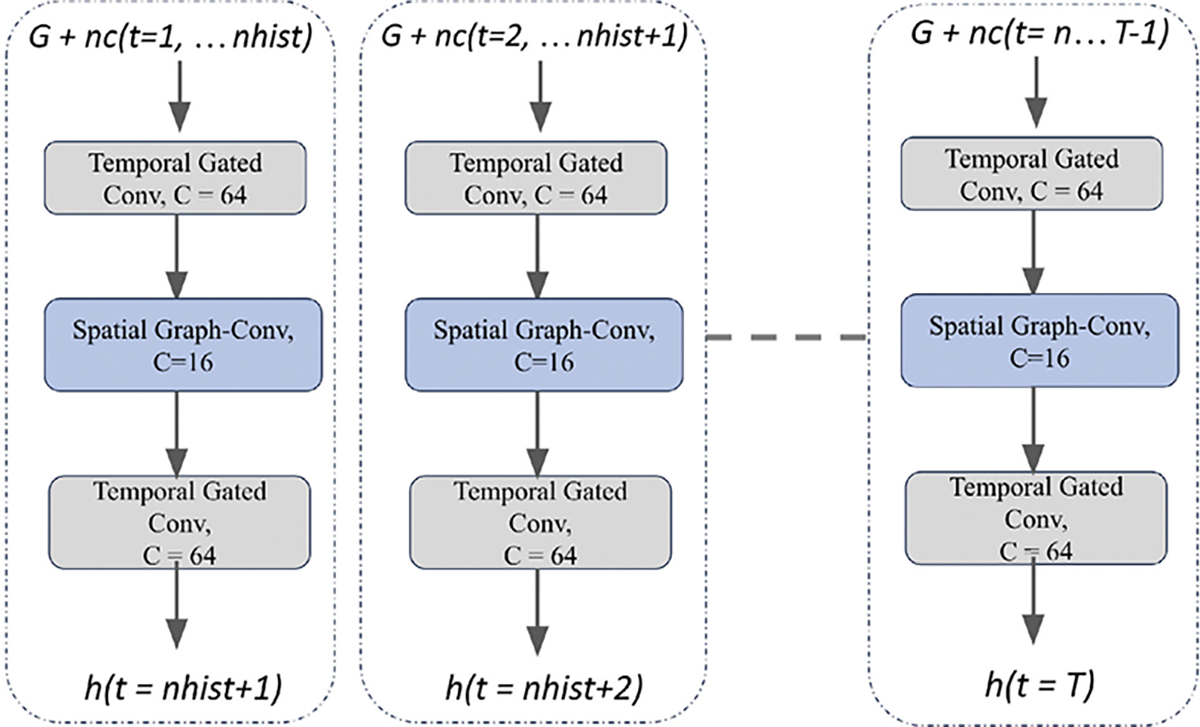
Architecture of the STGCN’s spatio-temporal convolutional (ST-Conv) block. Each ST-Conv block includes two temporal gated convolution layers and a spatial graph convolution layer, utilizing residual connections and bottleneck strategies to capture spatial and temporal dependencies.

After utilizing the active learning algorithm 2, our model can forecast the vaccine hesitancy for unknown ZIP code areas with just 18% of ZIP code information. Even with a small budget of B around 7% of the ZIP codes, the accuracy improves by 15% while predicting vaccine hesitancy for *T* + 1^*th*^ timestep, in comparison to the randomly selected nodes as shown in Figure 6. Although Figure 6 demonstrates one choice of B, we experiment with a wide range of B values. We find that increasing B improves the performance of VaxHesSTL, while decreasing it has the opposite effect.

In vaccine hesitancy, this combined approach with active learning is beneficial because it allows us to make forecasts for the entire network using data from a smaller subset of ZIP codes.

## V. CONCLUSION

Childhood diseases, such as measles, are now viewed as an imminent global threat [3]. Predictions of vaccine hesitancy at higher spatial resolution are highly useful for public health agencies in targeting interventions to improve immunization rates.

The VaxHesSTL framework is able to predict the spatio-temporal aspects of vaccine hesitancy using a combined GNN and RNN structure. This is a novel approach to studying vaccine hesitancy at a finer spatial scale. Our method outperforms several baseline methods in predicting vaccine hesitancy by leveraging a large database of healthcare claims, specifically the All-Payer Claims Database (APCD), collected from both public and private insurance providers. We also incorporate an aggregated ZIP code-level contact network derived from a detailed activity-based, individual-level population network. Our experiments find that closeness in the aggregated contact network has greater potential for predicting vaccine hesitancy compared to geographic closeness or physical distance (Table 3).

We utilize Moran’s-I and isolation indices [22] for quantifying spatial influence in the data. Insurance claims data exhibit higher Moran’s-I and lower isolation index values, indicating higher clustering and lower segregation in vaccine hesitancy. Our ablation study in the VaxHesSTL framework also shows the importance of the spatial component. Although LSTM is well-known to handle sequential data, we find that LSTM or MLP alone cannot predict vaccine hesitancy at a ZIP code level, since it does not exploit spatial correlations. However, a combination of GNN and LSTM can learn the spatial and temporal aspects of vaccine hesitancy and can predict patient refusal at a finer scale. We also demonstrate the model’s effectiveness at the node-level data, highlighting the challenges in learning vaccine hesitancy for smaller populations. We extend our framework by integrating active learning and demonstrate its capability to forecast even when vaccine hesitancy data is scarce or hard to get.

This research uses claims data aggregated at the ZIP code level. Although healthcare researchers extensively use claims data, they encounter several challenges, such as variations in coding practices among providers, overlap in the use of revenue and CPT codes, missing information, and discrepancies in claims costs [66]. Despite these challenges, the use of claims data in vaccine studies is increasing due to its richness [67]. Additionally, researchers have found that APCD data is consistent with medical report data [21]. In our research, we use ICD-10 codes, as our earliest data is from 2016, when the mandatory transition from ICD-9 to ICD-10 had already occurred. Furthermore, we only consider ZIP codes with a substantial population size to reduce data uncertainty. We note that there has been some other recent work on hesitancy using insurance claims and electronic health records [27], [45]; however, these have been limited to small datasets (less than 60K) with limited spatio-temporal resolution.

**Algorithm 3:** Data Transformation and Training Process for STGCN, $F\left(j,T+1,G,{nc}_{V}([1,T]),h_{V}([1,T])\right)$ for All j∈V

1:	**Input:** Data *data*, Model *STGNN*, Loss function *MSEloss*, Graph G, Number of historical timesteps *n_his*, Number of predictive timesteps *n_pred*
2:	**Data Transformation:**
3:	Initialize num_obs= total timesteps -n_his-n_pred
4:	**for** observation i in range(*num_obs*) **do**
5:	**for** timestep t in range(*n_his*) **do**
6:	Get the corresponding data for timestep $t\;{nc}_{V}([i,t])$ and $h_{V}([i,t])$ from *data*
7:	**end for**
8:	**end for**
9:	Split Dataset into train and test set
10:	**Training Loop:**
11:	Initialize *min_val _loss* = ∞
12:	**for** epoch in range(1, epochs +1) **do**
13:	*l_sum* = 0, *n* = 0
14:	**for** (x, y) in *train_data* **do**
15:	Get model predictions *y_pred* = *STGNN (x, G)*
16:	Reshape *y_pred* to match *y*
17:	Compute loss *l* = *MSEloss*(*y_pred, y*)
18:	Backpropagation: zero gradients, compute gradients, and update weights
19:	*l_sum*+ = *l* × *y.shape*[0]
20:	*n*+ = *y.shape*[0]
21:	**end for**
22:	Compute training loss: *train_loss* = *l_sum/n*
23:	**end for**

Our paper confirms the importance of spatial and temporal signals in an aggregated contact network for predicting vaccine hesitancy at a finer spatial scale. We also present an effective active learning approach to address the limited resources for acquiring additional spatial data, which is a common challenge in public health studies.

## Figures and Tables

**TABLE 1.** Notation used and their meaning.

Notation	Description
VaxHesSTL	Our Framework
F	The VaxHesitancy problem
G	Graph of relationships between ZIP codes
V	Set of all ZIP codes, denoted as the nodes of graph G
N	Number of ZIP codes
w(i,j)	Weight between ZIP code i and j, based on graph type.
$G_{a}$	Aggregated ZIP code level contact network
$G_{b1}$	Geographic adjacency graph (= first baseline graph)
$G_{b2}$	Distance-based graph (= second baseline graph)
$M_{s}$	Spatial module of VaxHesSTL
$M_{t}$	Temporal module of VaxHesSTL
$p_{i}(t)$	Number of children of age 0–6 years at ZIP code i at time t
nc(t)	Features at t time
$h_{i}(t)$	Vaccine hesitancy of i ZIP code at time t from data
T+1	Target time step for forecasting vaccine hesitancy
K	Subset of V used in training
$K^{\prime }$	Subset of V used in training for active learning
B	Budget for selecting ZIP codes to label for active learning
$\hat{y(t)}$	Output of the $M_{s}$ module for K ZIP codes, at time t
$\hat{h_{K}(t)}$	Output of the $M_{t}$ module for K ZIP codes, at time t
$V_{L}$	Nodes with Large Prediction Error
$V_{s}$	Nodes with Small Prediction Error

**TABLE 2.** Selected features from the APCD data.

Feature Name	Description
Incurred Date	The date of service (DOS)
MI Person Key	Member’s unique identification key
Member Zip Code DOS	Member’s ZIP code on the date of service
Member Age DOS	Member’s age on the date of service
Member Gender	Male, Female and Non-binary
Member Race	1 = American Indian/Alaska Native; 2 = Asian; 3 = Black/African American; 4 = Native Hawaiian/Other Pacific Islander; 5 = White; 6 = Unknown/Not Specified; 9 = Other; 0 = Unknown/Not Specified; Unknown = Unknown/Not Specified
Hispanic Indicator	01 = Yes; 02 = No; 03 = UNKNOWN
Payer LOB	Payer’s Line of Business: COMERCIAL, MEDICARE, or MEDICAID

**TABLE 3.** Average prediction performance of VaxHesSTL across three graphs connectivity mechanisms for T+1 Timestep in the test set.

Graph	MAE	MSE	MAPE	RMSE
Aggregated contact Graph $G_{a}$	**0.0275**	**0.00262**	**0.29773**	**0.0512**
Geographic Adjacency Graph $G_{b1}$	0.03058	0.00383	0.3766	0.0619
Distance-based Graph $G_{b2}$	0.0282	0.00291	0.34732	0.05402

**TABLE 4.** A comparative analysis between VaxHesSTL and baseline methods for forecasting, as well as the results of the ablation study. A higher $R^{2}$ value and lower values for MAE (Mean Absolute Error), MSE (Mean Squared Error), MAPE (Mean Absolute Percentage Error), and RMSE (Root Mean Squared Error) are preferred.

	T+1					T+4				
Method	$R^{2}$	MAE	MSE	MAPE	RMSE	$R^{2}$	MAE	MSE	MAPE	RMSE
VaxHesSTL	**0.8796**	**0.0275**	**0.0026**	**0.2977**	**0.0512**	**0.8498**	**0.0304**	**0.0034**	**0.3276**	**0.0580**
GNN-GRU	0.8591	0.0303	0.0031	0.3156	0.0555	0.8215	0.0328	0.0039	0.3518	0.0624
GCN-LSTM	0.7539	0.0368	0.0056	0.3872	0.0748	0.7446	0.0372	0.0056	0.3919	0.0747
LRN	0.6881	0.0454	0.0068	0.4887	0.0825	0.6669	0.0475	0.0072	0.5119	0.0853
MLP	0.7826	0.0362	0.0047	0.3899	0.0689	0.7024	0.0442	0.0065	0.4575	0.0802
GNN (VaxHesSTL w/o $M_{t}$)	0.8343	0.0315	0.0036	0.3518	0.0601	0.8358	0.0310	0.0036	0.3312	0.0598
LSTM (VaxHesSTL w/o $M_{s}$)	0.8094	0.0340	0.0041	0.3664	0.0645	0.7508	0.0388	0.0054	0.4185	0.0737

**TABLE 5.** Consistent performance of VaxHesSTL across different timesteps with a fixed time window $t^{\prime }=20$.

Training Time steps	Testing Time step	R^2^	MAPE
[1,20]	21	0.8596	0.363
[2,21]	22	0.8663	0.352
[3,22]	23	0.8734	0.338
[4,23]	24	0.8613	0.319

**TABLE 6.** Average values of significant features in the set $V_{L}$ (nodes with large errors) and $V_{S}$ (nodes with small errors).

Features	V_L_	V_S_
Children Population	311.62	873.24
Vaccine Hesitancy Percentage	0.026	0.033
Population Percentage with Medicaid	0.647	0.609
Hispanic Population Percentage	0.006	0.016

**FIGURE 8.**
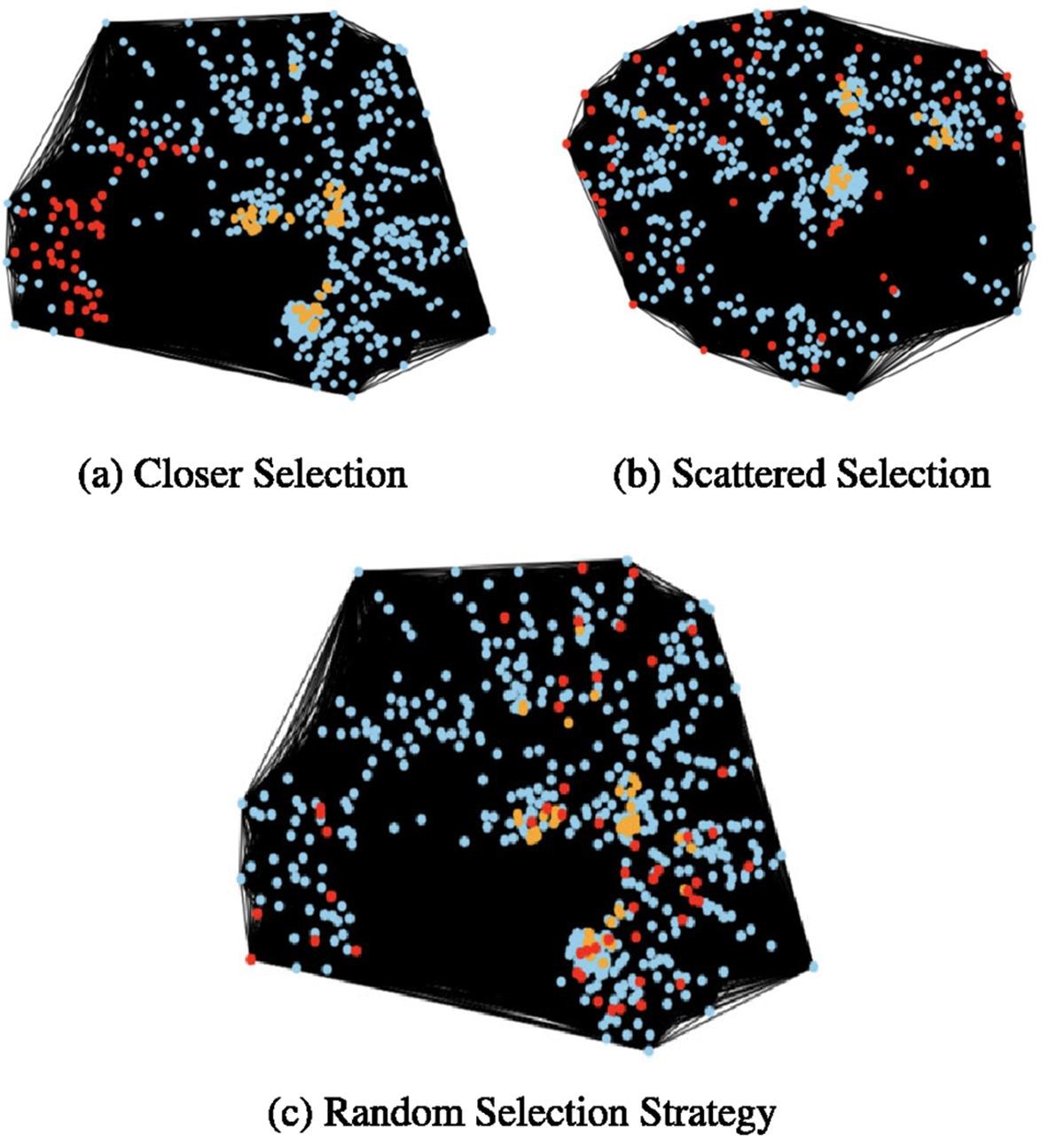
Three types of selection strategies are employed for initial training set selection. Nodes selected before Active Learning using these strategies are highlighted in red, while those chosen after employing active learning with VaxHesSTL are depicted in yellow.

**FIGURE 9.**
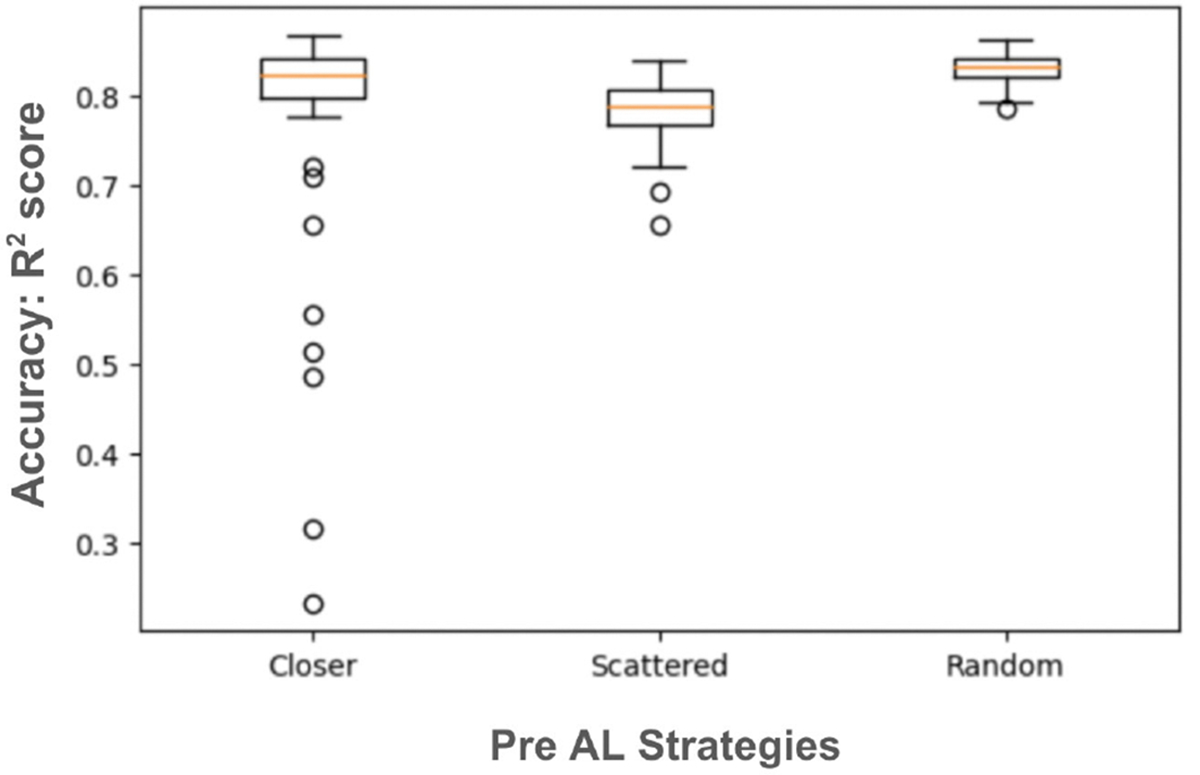
Accuracy comparison across various training set selection strategies preceding Active Learning with VaxHesSTL.

## APPENDIX A SCAN STATISTICS METHOD TO FIND THE UNDER-VACCINATED CLUSTERS

A cluster $C\subset G_{b1}$ of ZIP codes in the adjacent network $G_{b1}$ can have an arbitrary shape. We calculate the scan statistic or score function of a cluster of ZIP codes C as $F(C)=\frac{\mathrm{Pr}\left[\mathrm{Data}|h_{1}(C)\right]}{\left[\mathrm{Data}|h_{0}\right]}$ which is a likelihood ratio of the probability of the observed data (i.e., a certain level of vaccine hesitancy in C) generated under an alternative hypothesis $h_{1}(C)$, to the probability of the observations under the null hypothesis $h_{0}$. We use the Poisson version of the Kulldorff scan statistic. The null hypothesis $h_{0}$ is generated proportionally from the baseline count $\left(1-\mu )p_{i}(t\right)$, where μ is the state-wide vaccine hesitancy rate and $p_{i}(t)$ is population (0-6 years old) in a ZIP code i at time t. The alternative hypothesis of a cluster $h_{1}(C)$ counts the vaccine hesitancy among nodes outside C; in $V_{z}-C$, the hesitancy count comes from a rate proportional to the baseline counts. But, for the nodes within C, the counts are generated at a higher rate than expected. We use the Monte Carlo sampling approach to compute the p-value for each cluster [68]. Maximizing F(C) is the objective function. We use 0.9 as the cut-off p-value to consider significant clusters.

## APPENDIX B ALGORITHM AND FRAMEWORK FOR STGCN (SPATIO-TEMPORAL GRAPH CONVOLUTIONAL NETWORKS)

Spatio-temporal graph Convolutional Network (STGCN), illustrated in Figure 7, consists of multiple spatio-temporal convolutional blocks, each organized in a “sandwich” structure with two gated sequential convolutional layers and a spatial graph convolution layer in between.

Here, Graph CNNs are used to extract spatial features. Instead of segmenting the network into grids, this model uses graph convolution to leverage the full spatial structure, in our case the graph $G_{a}$. This approach provides more meaningful patterns by capturing both local and global relationships within the network.

To capture temporal dependencies, STGCN’s fully Convolutional Temporal Modeling can process vaccine hesitancy data over the time axis, removing the need for recurrent layers. This design enables efficient learning of temporal patterns.

## APPENDIX C ACTIVE LEARNING WITH DIFFERENT APPROACHES

### A. VISUALIZATION OF DIFFERENT STRATEGIES

We visualize the graph $G_{a}$ using NetworkX, where each node represents a Virginia ZIP code. Nodes are color-coded to distinguish between pre-AL strategies and selections following Active Learning (AL), offering insights into initial node selection across the static graph. The Pre-AL strategies, detailed in Section III-C, use a randomly chosen nodes approach, preferred over scattered and closer selections due to performance benefits. Figure 8 illustrates these pre-AL strategies on the graph, with red nodes representing Pre-AL selections and yellow nodes chosen iteratively through Algorithm 2.

Our VaxHesSTL is trained on the red and yellow nodes, enabling vaccine hesitancy forecasts across all ZIP codes.

### B. PERFORMANCE OF DIFFERENT PRE-AL STRATEGIES

In Figure 9, we present the results of multiple simulations where initial ZIP codes are selected using three different Pre-AL strategies. After creating the initial set of ZIP codes, we then apply the active learning method to identify and select additional important ZIP codes, allowing us to analyze and compare the performance outcomes across the different strategies. The plot illustrates that the randomly selected training set consistently outperforms the others prior to the active learning phase.

However, the closer strategy demonstrates occasional superior performance in certain iterations, suggesting that, in specific scenarios, focusing on nodes that are closely connected can yield better results. Overall, these findings emphasize the advantages of combining various selection methods to optimize node choice and enhance predictive accuracy in the active learning process.
